# A Rare Corticotroph-Secreting Tumor with Coexisting Prolactin and Growth Hormone Staining Cells

**DOI:** 10.1155/2012/529730

**Published:** 2012-12-17

**Authors:** Subramanian Kannan, Susan M. Staugaitis, Robert J. Weil, Betul Hatipoglu

**Affiliations:** ^1^Department of Endocrinology, Diabetes and Metabolism, Cleveland Clinic Foundation, 9500 Euclid Avenue Desk F20, Cleveland, OH 44195, USA; ^2^Departments of Anatomic Pathology and Neurosciences, Cleveland Clinic Foundation, 9500 Euclid Avenue Desk NC30, Cleveland, OH 44195, USA; ^3^Division of Brain Tumor and Neuro-Oncology Center, Cleveland Clinic Foundation, 9500 Euclid Avenue Desk ND40, Cleveland, OH 44195, USA

## Abstract

Pituitary adenomas can express and secrete different hormones. Expression of pituitary hormones in nonneoplastic pituitary cells is regulated by different transcription factors. Some pituitary adenomas show plurihormonal expression. The most commonly reported plurihormonal adenomas are composed of somatotrophs, lactotrophs, thyrotrophs and gonadotrophs. Pituitary adenomas composed of both corticotroph and somatolactotroph secreting cells are not common because transcription factors regulating the expression of these hormones are different. We report a rare case of pituitary adenoma with concomitant corticotroph, prolactin, and growth hormone staining cells, review literature on similar cases, and discuss possible biological mechanisms underlying these plurihormonal tumors.

## 1. Case Summary

**Figure 1:**
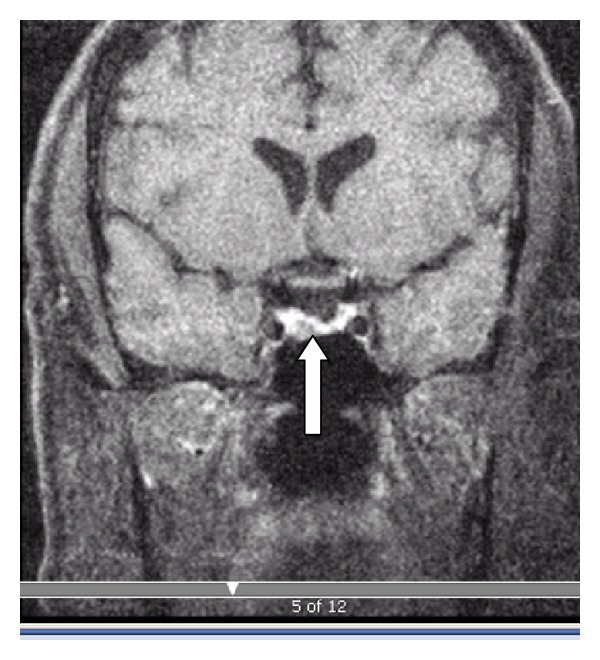
Pituitary MRI (post-contrast) showing microadenoma (thick arrow).

A 52-year-old grocery store manager presented with symptoms of fatigue, poor libido, and erectile dysfunction for a two-year period. Onset was insidious and some of his symptoms were partially relieved by testosterone gel that was prescribed to him by his primary care doctor. He noted a 50 lb weight gain in a period of 6 months that prompted him to see an endocrinologist. In addition to the fatigue, he noted increasing muscle pain and proximal weakness over the past 6 months. He complained of increased ring and shoe size but it was difficult to clarify whether this was related to his generalized weight gain or specifically to his acral enlargement. On review of symptoms, he noted easy bruisability but denied breast enlargement or galactorrhea. His hypertension was well controlled with two medications and was not diagnosed with glucose intolerance. Clinical exam revealed a morbidly obese male with BMI of 48. His obesity was truncal, and he had disproportionately thin proximal musculature. Abdomen exam revealed 1 cm purple striae. His facial features were suggestive of cushingoid appearance. He did not have any definitive physical signs of acromegaly. His preoperative labs are summarized in Table 1, which were was conclusive for Cushing's syndrome. He had mild elevation of prolactin (PRL), insulin-like growth factor (IGF-1) (Table 1). GH nadir after a 75 g glucose challenge was 0.36 ng/mL. MRI of the sella showed a pituitary microadenoma (5 mm) with a normal appearing stalk (Figure 1). Inferior petrosal sinus sampling confirmed the presence of pituitary source of ACTH hypersecretion. The patient underwent trans-sphenoidal resection of an unencapsulated tumor. The rest of the pituitary was carefully scrutinized with no evidence for additional tumors.

**Figure 2:**
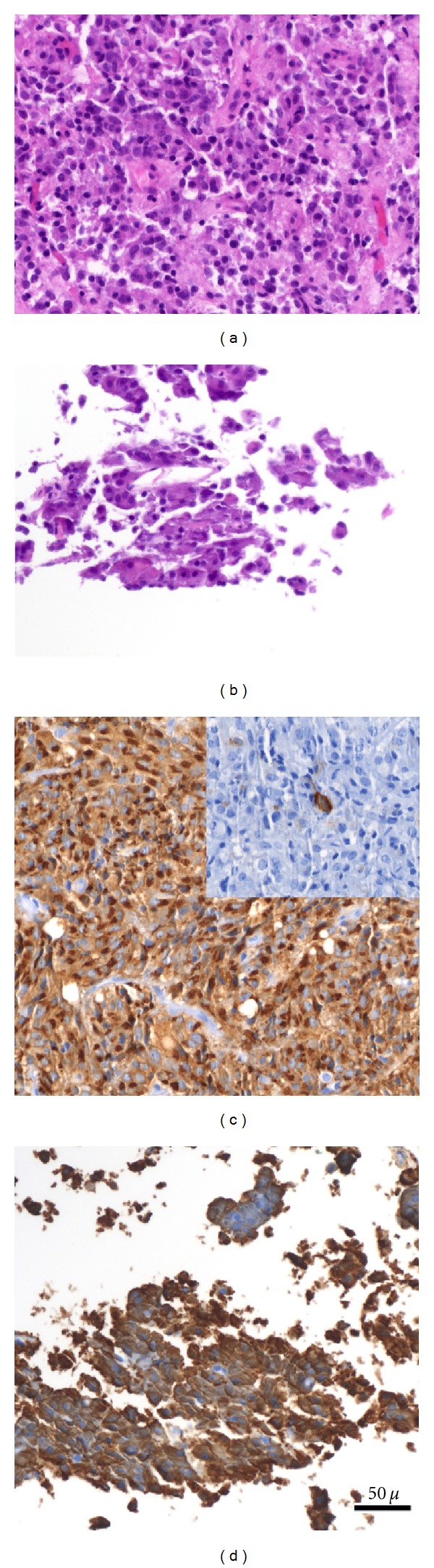
Pathology images of the tumor. (a) H&E stain of the larger tumor tissue showing abundant eosinophilic cytoplasm. (b) H&E stain of the smaller tissue showing abundant basophilic cytoplasm. (c) Immunohistochemistry: all of the cells in the larger adenoma were immunoreactive with antibodies to prolactin, and rare cells (Inset) were growth hormone positive. (d) Immunohistochemistry: cells of the small pieces of adenoma were ACTH positive.

Histology of the resected tissue revealed one piece of tissue measuring 1 mm^2^ and that was composed of an adenoma with abundant relatively eosinophilic cytoplasm (Figure 2(a)). In addition, there were several smaller pieces of adenoma each measuring 0.2 mm in greatest dimension. These cells had less abundant and more basophilic cytoplasm (Figure 2(b)). Immunohistochemistry was performed using antibodies to PRL, GH, ACTH, TSH, LH, and FSH. All of the cells in the larger adenoma were immunoreactive with antibodies to PRL (A0569, Dako), and rare cells were positive to GH (A0570, Dako, Figure 2(c)). The cells of the small pieces of adenoma were ACTH positive (RB-9217-P0, Thermo-Fisher, Figure 2(d)). There was no overlap between the prolactin-positive and ACTH-positive cells. No cells labeled with antibodies to FSH (M3504, Dako), LH (M3502, Dako), or TSH (112A-18, Cell Marque). The patient was supplemented with hydrocortisone postoperatively. His PRL and IGF-1 levels normalized (Table 2).

## 2. Discussion

Pituitary adenomas can express and secrete different hormones. Expression of pituitary hormones in nonneoplastic pituitary cells is regulated by different transcription factors. Pituitary-specific positive transcription factor 1 (PIT-1) regulates expression of GH, PRL, and TSH, and the steroidogenic factor 1 (STF-1) and endothelial transcription factor GATA 2 regulate expression of LH and FSH. ACTH expression is regulated by tumor homeobox transcription factor19 (TBX 19, also known as TPIT) [9]. Some pituitary adenomas show plurihormonal expression. The most commonly reported plurihormonal adenomas coexpress growth hormone (GH), prolactin (PRL), and/or thyroid stimulating hormone (TSH) or luteinizing hormone (LH) and follicle-stimulating (FSH) [10]. In view of the transcription factors that regulate the synthesis of the individual hormones, this finding is not surprising.

Plurihormonal tumors are either monomorphous (different hormones expressed from a single cell type or plurimorphous (different hormonal expression from different cells) [11–13]. Our review of the literature (English language only) revealed few case reports of plurihormonal tumors with concomitant presence of ACTH and PRL/GH staining tumor cells [1–8, 14]. Details of clinical presentation and staining pattern of eight cases are listed in Table 2. In contrast to the previously published cases, where the tumor was a macroadenoma and the predominant clinical presentation was acromegaly, our patient had a microadenoma and dominant clinical presentation was Cushing's disease.

Diagnosis of plurihormonal tumors relies on immunohistochemistry demonstrating significant and specific immunoreactivity to unrelated hormones using specific antisera. The case we report clearly had two histologically distinct populations of cells with evidence of different immunohistochemical hormonal expression. We have excluded the common pitfalls for misdiagnosis of plurihormonality. The histology of the specimen removed from the patient in our case showed only adenoma and did not include any tissue recognizable as entrapped normal adenohypophysis. The pattern of immunoreactivity for ACTH, PRL, and GH in the specimen was nonoverlapping and supports the interpretation that the antibodies used for detection were specific (Figure 2). It is impossible to conclude whether the expression of PRL and GH in one portion of the specimen influenced the clinical presentation of our patient. Although our patient did have marginally elevated PRL and IGF-1 levels, their clinical significance is uncertain.

The pathogenesis of plurimorphous plurihormonal tumors is hypothesized to be as a result of neoplastic transformation of two different cell lines or transdifferentiation of a once tumor cell line into a different hormone-producing cell line [9, 12, 15]. The possibility of tumors developing from noncommitted stem cells in the pituitary also has been suggested [15]. We did not perform an electron microscopy or a transcription factor analysis in our case, which limits further speculation on the original tumor cell line. Further work is required to elucidate the role of specific factors and the molecular mechanisms contributing to normal and abnormal pituitary development.

## Figures and Tables

**Table 1:** Hormonal profile pre- and postoperatively.

Test	Normal range	Preoperative	Postoperative
Testosterone (Total)	300–890 pg/mL	91	195
Testosterone (Free)	47–244 pg/mL	17	NA
Cortisol (AM)	3.4–26.9 mcg/dL	19.7	3.2
ACTH (AM)	8–42 pg/mL	80	28
IGF-1	61–285 ng/mL	323	241
Prolactin	2.1–17.7 ng/mL	18.9	7.3
Free T4	0.8–1.8 ng/dL	1.2	1.3
Cortisol (LDDST*)	<1.8 mcg/dL	10.8	<1.0
Salivary cortisol (midnight)	<50 ng/dL	114	
GH (Basal)	0.01–0.97 ng/mL	0.20	<0.01
GH (75 g glucose load—30 min)		0.21	
GH (75 g glucose load—60 min)		0.46	
GH (75 g glucose load—120 min)		0.36	

*LDDST: Low-dose dexamethasone suppression test.

**Table 2:** Case reports of concomitant ACTH and GH/PRL secreting tumors described in the literature.

Author and year	Age/sex	Size of adenoma	Dominant clinical presentation	Acromegaly evaluation	Cushings evaluation	Pattern of stains
Arita et al. 1991 [1]	29/F	1.6 cm	Acromegaly and Cushing's	GH 92 ng/mL	ACTH 94 pg/mL	Diffuse GH stainings and focal ACTH staining in the tumor tissue
Blevins et al. 1992 [2]	40/F	1.3 cm	Acromegaly and Cushing's	GH 22 ng/mL Nonsuppression of GH with OGTT	UFC 731 nmol/d (66–298) Nonsuppression of UFC with 2 mg Dexamethasone	Two discrete cell groups each staining diffusely for GH and ACTH, respectively
Apel et al. 1994 [3]	76/F	2.2 cm	Acromegaly	GH 13.7 ng/mL (<5) IGF-1 464 ng/L (116–270)	ACTH 6.5 pmol/L (<22) PRL 25.8 mcg/L (<17.9)	Two discrete areas of GH and ACTH staining
Kovacs et al. 1998 [4]	62/M	>1 cm**	Acromegaly	GH 13.7 ng/mL (<5) IGF-1: 973 ng/L (48–275)	Not available	Majority of cells displayed variable positivity for GH, whereas the minority of adenoma cells were strongly stain for ACTH; occasional cells positive for PRL
Mazarakis et al. 2001 [5]	53/M	1.5 cm	Acromegaly	GH 17.5 ng/mL IGF-1 934 ng/mL	ACTH 29 pg/mL (<52) No hypercortisolism documented	Chromophobic tumor, immunoreactive for GH focally admixed with hyperplastic PAS positive, and ACTH immunoreactive cells showing the electron microscopic features characteristic of corticotrophs
Kageyama et al. 2002 [6]	45/F	1.2 cm	Acromegaly	GH 20.9 ng/mL (<3.1) IGF-1 650 ng/mL (46–282)	ACTH 91 pg/mL (<60) Urine-free cortisol 403 mcg/d (40–140) LDST-Cortisol Nonsuppression HDST-cortisol suppression	2 distinct types of tumor cells, with either diffuse but faint GH-positive cells or sparse but distinct ACTH-stained cells; few PRL positive cells detected as well
Tsuchiya et al. 2006 [7]	54/M	1.2 cm	Acromegaly	GH: 31.5 ng/mL (<0.6) IGF-1: 493 ng/mL (106–398)	ACTH: 53 pg/mL (7–56) UFC: 45.6 mcg/d (<80.3) LDST-Cortisol Nonsuppression HDST-cortisol suppression	Diffuse GH and PRL staining and focal ACTH staining in the tumor tissue
Oki et al. 2009 [8]	36/M	>1 cm**	Acromegaly	GH 6.33 ng/mL IGF-1: 1361.3 ng/mL (67–318)	ACTH 91.7 pg/mL LDST-Cortisol Nonsuppression HDST-cortisol suppression	Tumor cells with diffuse strong GH stain and focal staining with ACTH and TSH

**Clear evidence of macroadenoma on the MRI images, size details not provided in the case reports.

Values in parenthesis indicate normal ranges.

UFC: urine-free cortisol.

LDST: low-dose dexamethasone test (0.5 or 1 mg).

HDST: high-dose dexamethasone test (8 mg).
